# Orbital Apex Syndrome secondary to residual tooth root infection: a case report^☆^

**DOI:** 10.1016/j.bjorl.2024.101455

**Published:** 2024-06-10

**Authors:** Zongxuan He, Hongyu Han, Wei Shang, Kai Song

**Affiliations:** a The Affiliated Hospital of Qingdao University, Department of Oral & Maxillofacial Surgery, Shandong Province, China; b Qingdao University, School of Stomatology, Shandong Province, China; c Qingdao University, Qingdao Municipal Hospital, Shandong Province, China

## Introduction

Orbital Apex Syndrome (OAS) refers to a group of rare disorders characterized by lesions infiltrating the tissues of the orbital apex region, resulting in impairment of cranial nerves II, III, IV, V, and VI. The Clinical symptoms include orbital pain, subtle proptosis, ptosis, disturbances in eye movement, sluggish pupillary light reflex, and progressive sight loss, potentially leading to blindness.^1^ The etiology of OAS is frequently associated with trauma, tumors, and autoimmune diseases. Although infections arising from the paranasal sinuses and periorbital region have been reported to result in OAS,^2^ odontogenic infections are likely to be ignored.

## Case report

**Figure 1:**
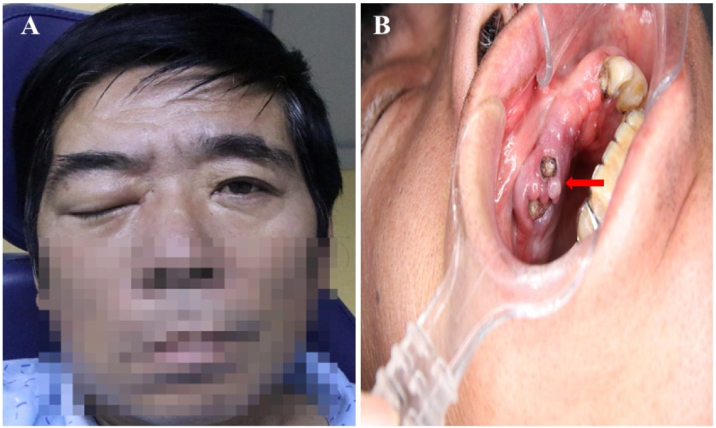
Clinical features at presentation. (A) Complete ptosis of the right eyelid. (B) Redness and swelling of the mucosa around the residual roots (red arrow).

**Figure 2:**
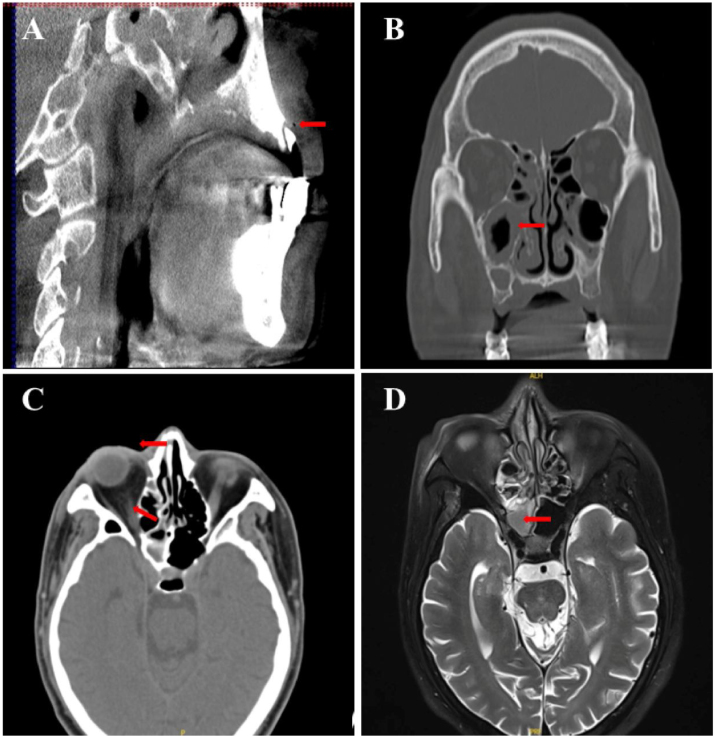
Computed tomography images. (A) Apical periodontitis in the maxillary anterior teeth. (B) Concurrent maxillary and ethmoid sinuses. (C) Right optic perineuritis with proptosis. (D) MRI showing prominent inflammation of the right maxillary and ethmoid sinuses.

A 58-year-old man, with inadequately controlled diabetes *mellitus* (HbA1c = 12.1%), presented initially to the Department of Neurology with the sudden onset of diminished visual acuity in the right eye and the ptosis of the right eyelid (Fig. 1A). Laboratory results showed a white blood cell count of 15.38 × 10^9^/L (83.1% neutrophils) and a C-reactive protein level of 46.61 mg/L. The fasting blood glucose levels (10.5 mmoL/L) and urinary analysis showed 4+ glucose, accompanied by 2+ ketones. The brain MRI and orbital CT scans revealed mucosal thickening in the nasal sinuses, peri-orbital swelling, and protrusion of the right eyeball, with no anomalies identified within the cranial cavity (Fig. 2). The patient had a 10-year history of poorly controlled type 2 diabetes *mellitus*, therefore, considering clinical manifestations and laboratory examinations, the preliminary diagnosis was severe multiple cranial nerve impairments secondary to sinusitis and diabetes *mellitus*. Two days later, the antimicrobial regimen was switched to vancomycin based on the results of bacterial cultures and susceptibility testing of nasal secretions. Unfortunately, there was still no marked improvement in the patient’s condition for one week.

Subsequently, the case was discussed in a multidisciplinary team meeting, involving specialists from the fields of otolaryngology, neurological surgery, oral and maxillofacial surgery, and ophthalmology. The patient had a history of multiple tooth loss due to periodontitis, with only two residual tooth roots on the right side of the maxilla. Ten days prior, the patient experienced right maxillary tooth pain accompanied by right facial pain and swelling, without specific treatment. Examination revealed tenderness upon percussion in the residual root of the right upper canine region, accompanied by gingival redness and swelling, vestibular groove swelling, and tenderness upon palpation in the right facial region.

**Figure 3:**
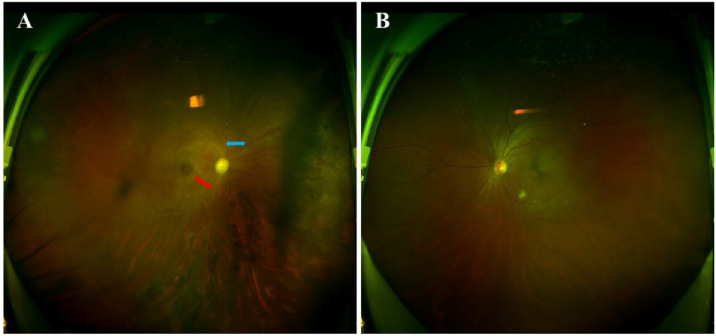
Fundi examination images. (A) Central retinal artery occlusion and cherry-red spot (red arrow), optic nerve atrophy (blue arrow) in the right eye. (B) Normal left eye.

Considering the presence of residual roots in the upper right maxilla along with periapical periodontitis (Fig. 1B), the diagnosis leaned to-wards “right OAS induced by periapical periodontitis”. Sub-sequent interventions included extraction of residual roots in the upper right maxilla, sinus fenestration and drainage performed by an otolaryngologist, and proactive blood glucose control. The patient's condition eventually stabilized after one week. However, complete restoration of the visual acuity was not achieved. Ophthalmological investigations revealed central retinal artery occlusion and optic atrophy (Fig. 3). At the one-month follow-up, the patient's infectious symptoms had subsided, and vision in the right eye was light perception, while that in the left eye was normal.

## Discussion

Odontogenic infections leading to OAS are rare and pose diagnostic challenges. Oral and maxillofacial surgeons are often unaware of condition. The mechanism underlying blindness resulting from OAS due to odontogenic infections remains unclear. Here, we posit that the prolonged elevation of the patient's blood glucose level initially resulted in heightened fragility of the retinal vessels, rendering them more prone to damage.^3^ Second, in cases of secondary periorbital infection caused by the affected tooth, minute bacterial emboli detach, obstruct the central retinal artery, and lead to visual impairment. Furthermore, recovery is challenging in the presence of blindness.

The pathogens responsible for infections in the orbital apex are diverse.^4^ Reports suggest that fungal infections are generally prognosticated less favorably than their bacterial counterparts.^5^ Therefore, it is imperative to conduct bacterial cultures and sensitivity tests as early as possible. In this case, Methicillin-Resistant Staphylococcus Aureus (MRSA) was identified in the patient's nasal sinus secretions, and prompt replacement with vancomycin was beneficial. The use of advanced antibiotics, such as vancomycin, as an initial treatment is not recommended. Nevertheless, timely and robust therapeutic interventions are necessary for patients with compromised immune functions.

Our treatment experience was similar to that described in Leferman’s review.^5^ However, the population-based incidence of odontogenic maxillary sinusitis remains unknown. Retrospective investigations suggest that odontogenic maxillary sinusitis may contribute to 25%–40% of all cases of chronic maxillary sinusitis in the Asian/Chinese population. Although maxillary sinus infections following maxillary molar extraction are common, the development of OAS due to persistent apical periodontitis in the maxillary canines has not been previously reported. We hypothesized that apical infections initially affect the maxillary sinus before spreading to other sinuses. The orbital apex region may communicate freely with the sinuses and cavernous sinuses through its valveless blood vessels, thereby facilitating the dissemination of the infection.

In cases of OAS resulting from odontogenic infections, apart from conventional anti-infective therapy, we advocate a proactive stance, particularly when imaging reveals abscess formation, thereby endorsing early incision and drainage. Prompt initiation of multidisciplinary interventions is paramount. The prompt initiation of treatment prevents the progression of harmful sequelae.

## Conclusion

OAS is a complex clinical syndrome and cases attributing this severe condition to odontogenic infections have seldom been documented. This case report aimed to enhance the understanding of oral and maxillofacial surgeons regarding OAS. Timely sinus surgery involving window drainage and extraction of the affected teeth may mitigate disease progression.

## Ethics approval and consent to participate

Ethics approval for this case report was not required according to the guidelines stated by the Research Ethics Board of Qingdao University.

## Conflicts of interest

The authors declare no conflicts of interest.
